# Bridging policy and implementation science: operationalising political and structural determinants for health policy implementation evaluation

**DOI:** 10.1186/s43058-026-01137-0

**Published:** 2026-10-11

**Authors:** Sarah Forberger, Biljana Meshkovska, Lucia A. Reisch, Aleksandra Luszczynska, Hajo Zeeb

**Affiliations:** 1 Leibniz Institute for Prevention Research and Epidemiology- BIPS, Achterstrasse 30, 28359 Bremen, Germany; 2 El-Erian Professor of Behavioural Economics and Policy, University of Cambridge, Cambridge, CB3 9ET UK; 3 Department of Nutrition, Institute of Basic Medical Sciences, Faculty of Medicine, University of Oslo, Domus Medica, Sognsvannsveien 9, Oslo, 0372 Norway; 4 Institute of Psychology, SWPS University, Ostrowskiego Street 30b, Wroclaw, PL53238 Poland; 5 Lyda Hill Institute of Human Resilience, University of Colorado at Colorado Springs, 4863 North Nevada Avenue, Colorado Springs, CO 80918 USA; 6 Health Sciences Bremen, University of Bremen, Bibliothekstr. 1, 28359 Bremen, Germany

**Keywords:** Policy implementation, Public health policy, Implementation science, Political determinants, Structural determinants, CFIR, CICI, Implementation evaluation, Operationalisation

## Abstract

**Background:**

Despite their critical role in shaping public health policy implementation, key political and structural determinants within implementation frameworks are often poorly defined, underreported, or treated as background rather than as core implementation variables. Existing implementation science frameworks acknowledge these determinants, they lack systematic, operationalised guidance for reporting them, resulting in limited explanatory power, poor comparability, and an overemphasis on individual-level factors.

**Objective:**

This paper aims to improve reporting standards in public health policy implementation evaluation by defining key political and structural determinants and proposing their operationalisation within established implementation frameworks.

**Methods:**

The synthesis drew on seven systematic and scoping reviews and three in-depth case studies from the PEN, ASTRA, and STOP projects, covering nutrition, physical activity, and tobacco control. We consolidated predefined, theory-informed extraction categories from the underlying reviews, together with recurring reporting gaps, into overarching reporting dimensions. We then contextualised and refined these dimensions using evidence from the three case studies across diverse governance levels and socio-political settings.

**Results:**

We consolidated and operationalised eight core reporting dimensions: (1) policy objectives and scope, (2) actors, power, and competence, (3) legal status and jurisdiction, (4) policy instruments (regulatory, economic, communicative, behavioural), (5) implementation strategies (centralised vs. decentralised, coordination mechanisms), (6) resources (financial, human, material, and implementation costs), (7) equity impact and (8) policy lifecycle. The dimensions specify political and structural information relevant to policy implementation evaluation and provide a structured basis for more systematic and transparent reporting across diverse policy contexts. The operationalisation is designed to complement and to be integrated into established implementation frameworks.

**Conclusion:**

Systematic and transparent reporting of political and structural determinants is essential for understanding the success or failure of policy implementation. The proposed operationalisation offers researchers and evaluators a practical approach to strengthen public health policy implementation evaluations. Designed for integration into frameworks such as CFIR and CICI, it may help identify leverage points, support adaptive policy design, and contribute to more effective, equitable, and sustainable public health interventions.

**Supplementary Information:**

The online version contains supplementary material available at https://doi.org/10.1186/s43058-026-01137-0.

Contributions to the literature

- Bridges implementation science and policy implementation research by translating political and structural determinants into operationalised reporting dimensions.
- Builds on existing frameworks, including CFIR and CICI, by specifying policy-level information on actors and power, legal authority, policy instruments, implementation arrangements, resources, equity, and policy lifecycle.
- Addresses persistent reporting gaps by promoting more systematic and transparent documentation of political and structural determinants.
- Supports more comparable and context-sensitive evaluations of public health policy implementation, while highlighting the need for further validation across policy and governance settings.

## Introduction

Public health policies have driven major public health advances including vaccination programmes, motor-vehicle safety measures, improved workplace conditions [1], smoking bans, sugar-sweetened beverage taxation, and marketing regulations for unhealthy foods [1–4]. These approaches underscore the transformative potential of policy-driven changes. With the rise of non-communicable diseases (NCDs), there is a growing emphasis on addressing NCDs through the living environment, the physical, institutional, economic, and cultural context in which people make decisions about their lifestyle [1].

Many NCD risk factors, such as tobacco use, unhealthy diets, and physical inactivity, are strongly shaped by individual, physical, and societal structures [1, 5]. Individual prevention assumes people have the necessary resources, competencies, and motivation to initiate and maintain behaviour change, yet personal constraints and structural inequalities rooted in poverty, education, and living or working conditions often limit its success. These disparities are not country-specific. They appear across countries and often stem from historical national, state, and local policies of intentional disinvestment and oppression, as well as policies that have produced unintended inequitable consequences for specific groups. Accordingly, presenting structural context as apolitical or neutral can be reductive, as historical policies continue to shape living environments that produce physical and social inequities through path dependence, anchoring effects, and historical lock-ins. These influences are rarely acknowledged, monitored, or actively counteracted, and they may reinforce past patterns and perpetuate inequities [6–8]. Because individual behaviour and structural context shape each other dynamically, sustainable behaviour change requires not only individual agency but also the intentional redesign of the environments in which people live and work, through policies that address both individual and structural inequalities [9–19]. Conversely, policy initiatives must, in turn, align with the behavioural realities of those they aim to influence [20].

Public policies are a primary mechanism for such environmental change. Typically introduced by governments, they aim to reshape living environments to target changes in individual behaviour and ultimately alter social norms [21]. Examples include altering planning regulations, such as specifying the width of cycling and pedestrian pathways, community catering standards, labelling requirements, and taxation on alcohol, tobacco, or sugar-sweetened beverages. Although such policies typically acknowledge marginalised and/or minoritised groups, they rarely account for path dependencies, anchoring effects, and historical impacts, and therefore do not include them in policy design. For example, SSB taxes and active transport policies risk disproportionately burdening low‑SES/minority communities due to limited access to healthy alternatives and infrastructure, if not well designed and monitored [22–25]. Understanding whether and how such policies achieve their intended effects therefore requires attention not only to policy design but also to the processes and contexts of implementation.

Implementation science developed largely within medicine and the evidence-based practice movement, which focuses on clinical settings and intervention fidelity [26] and has historically prioritised individual-level determinants, such as provider attitudes or patient adherence, while underreporting or under-theorising the political, institutional, and structural conditions that enable or constrain policy uptake and sustainability [21, 27]. These foundations offer valuable insights but do not readily transfer to public policy implementation, which operates within distinct institutional logics, political dynamics, and governance constraints, and involves government-led actions aimed at systemic change across multiple governance levels [28–35]. To advance the field, therefore, requires an integrated approach that considers individual-level, political, institutional, and structural determinants together.

The complexity of public policy implementation evaluation arises from the systems in which policies operate, individual and structural determinants, the involvement of diverse stakeholders at every level, interactions between levels, and the absence of clear reporting guidelines [36–43].

Despite their potentially transformative power, policy evaluations remain scarce, and findings often lack sufficient depth to inform practice or enable meaningful comparisons, undermining learning, replication, and the development of evidence-informed policy strategies. Frameworks such as the Context and Implementation of Complex Interventions (CICI) and the Consolidated Framework for Implementation Research (CFIR) offer valuable guidance for organising implementation evaluation [40]. However, they often lack systematic and detailed guidance on operationalising political and structural determinants, such as legal status, policy instruments, stakeholder power, and jurisdictional complexity. They provide limited guidance on how to specify, measure, and interpret these factors in relation to implementation outcomes. This gap is particularly evident in the under-theorisation of “policy” itself, which is frequently treated as context, a facilitator, or a barrier, rather than as a core policy implementation factor [41, 44].

The article therefore aims to operationalise political and structural determinants by starting with the task of operationalising “…exactly what policy is and does.” [45]. As Crable et al. and Chriqui et al. note, the explicitness of “policy” within dissemination and implementation science is frequently underdefined and, at times, obscured [41, 44]. Crable et al. propose six dimensions for strengthening the operationalisation of “policy” within frameworks such as EPIS (exploration, preparation, implementation, and sustainment), specifying the dimensions of a policy’s function, its form, defining the nonlinear phases of policy dissemination and implementation, describing stakeholders’ temporal role, accounting for inner and outer context adaptations, and identifying bridging factors essential for success [41]. McLoughlin et al. highlight the need for equity-informed, policy-specific implementation measurement, arguing that policy implementation science studies have received disproportionately little attention despite offering substantial potential to address health disparities [46].

Purtle et al. emphasise treating policy not merely as a contextual variable but as an object of rigorous conceptualisation and reporting. This entails systematically documenting how policy choices are theorised within implementation research and how operational definitions are derived and publicly reported [47]. Bullock et al. detail how policy is used across implementation science in different health fields and its connection to policy theory, as context, innovation, enabler, or barrier, and as an outcome. However, they acknowledge that the field still lacks scalable strategies for operationalising these insights across diverse implementation frameworks [48].

Campos et al. complement this perspective by analysing stakeholder groups and political dynamics that can shape policy implementation, including interest groups, bureaucrats, budget politics, leadership politics, beneficiary politics, and external actors [49]. While Campos et al. focus on how dynamics within stakeholder fields influence health policy implementation, Bullock et al. differentiate specific actor groups [48, 49]. This aligns with Allen et al., who systematically analysed how health policy implementation is measured to identify implementation outcomes [50]. The most commonly assessed implementation outcomes were policy fidelity and compliance with the government mandate, acceptability of the policy among implementers, perceived appropriateness of the policy, and implementation feasibility. However, they also note a lack of attention to how a policy aligns with the external implementation context and to the policymaker’s role, arguing that the most essential determinants of policy implementation are not well known [51–53]. These points have direct implications for designing policy implementation evaluations. Smith et al. recommend specifying the policy’s implementation logic, assembling an interdisciplinary team, and using mixed methods to identify, measure, and analyse relevant determinants of policy implementation and the processes involved. They also call for incorporating flexibility into project timelines to accommodate delays and challenges inherent in the real-world nature of policy [54].

All authors call for operationalising the “objective in question” [55]. Public policy research reveals a similar analytical gap. The four major policy process theories have distinct focal points. The Advocacy Coalition Framework emphasises the role of coalitions with shared beliefs in driving policy change over time. The Multiple Streams Framework highlights how policy changes occur through the confluence of problems, politics, and policy streams, creating windows of opportunity. The Narrative Policy Framework focuses on how narratives shape and influence policy debates, and the Punctuated Equilibrium Theory examines periods of stability disrupted by sudden policy changes [56]. All these aspects are crucial for implementation in and of themselves. However, while they focus on political dynamics, decision-making processes, and policy windows, they often neglect how policies are implemented and which instruments are used to achieve policy outcomes. Understanding these elements is crucial for assessing policy processes, their practical implications, and their effectiveness [57–61].

### Research question

Building on these foundations, this study addresses the following questions:

1. How can the systematic and practical reporting of political and structural determinants in health policy implementation be enhanced through operationalisation informed by public policy research?
2. How can operationalisation and guiding questions complement established implementation science frameworks, such as CFIR or CICI, to improve the consideration and reporting of political and structural determinants?

This paper aims to serve as a practical guide for strengthening policy implementation evaluation. It seeks to stimulate further research, raising awareness of the critical role of political and structural context in implementation. The goal is not to replace existing frameworks but to integrate the strengths of implementation science and policy analysis to create a robust and transparent approach.

## Methods

This study used a theory-guided, integrative synthesis to develop an operationalisation for systematically considering and reporting political and structural determinants in health policy implementation evaluations. It integrates conceptually related research conducted by the author team over the past seven years across several policy domains and implementation contexts. The synthesis is built on predefined, theory-informed extraction categories and recurring reporting gaps identified across the underlying reviews, and empirical insights from in-depth case studies. Drawing on findings from seven systematic and scoping reviews (Table 1) and three case studies covering the EU School Fruit and Milk Scheme (across 10 European countries), physical activity policy implementation in elementary schools in Baden-Württemberg, Germany, and tobacco control in Khyber Pakhtunkhwa, Pakistan, we integrated evidence from multiple projects (PEN, ASTRA, STOP) across nutrition, physical activity, and tobacco control domains (Table 1, Appendix 1).

The underlying research activities shared an interest in policy implementation and the influence of political and structural contexts. The implementation-focused scoping reviews were closely aligned in their overarching interest in political and structural determinants, while the systematic reviews complemented this work by providing evidence on policy effectiveness and on existing evaluation frameworks. The case studies were subsequently used to examine, contextualise and refine the emerging operationalisation.

The rationale for the present study emerged from a recurring methodological challenge observed throughout this research. Political and structural determinants are widely recognised as potentially important influences on policy implementation. However, they are not consistently defined and operationalised in implementation research and may, therefore, not be systematically considered during the design, conduct, or reporting. The absence of reporting on a political or structural determinant, however, cannot be interpreted as evidence that the determinant was absent from the policy context or did not influence implementation.

This creates a persistent evidence dilemma, making it difficult to determine whether they were absent, unexamined, or present but unreported. Repeated observations of this dilemma across our research activities provided the conceptual impetus for the present study. The aim was therefore to develop an operationalisation that enables political and structural determinants to be systematically considered and reported, rather than inferred retrospectively from incomplete information.

The reviews and case studies were conducted independently within their respective research projects and retained their original methodological designs. In the present study, they were brought together at a higher conceptual level because of their complementary contributions to understanding policy implementation and recurring reporting gaps.

The three scoping reviews provided evidence on determinants, barriers, facilitators, and implementation processes. The systematic reviews complemented this evidence by examining policy effectiveness and the approaches and frameworks used for implementation evaluation. Together, these reviews allow us to compare what is known about policy effectiveness and implementation with what is actually reported about the political and structural contexts in which implementation takes place. The case studies provided detailed, context-specific evidence from diverse policy domains, governance levels, and socio-political settings, and we used them to assess whether the emerging determinant categories and reporting dimensions were meaningful and applicable across heterogeneous implementation contexts, and to refine the resulting operationalisation (Appendix 1). Across the reviews, recurring methodological observations emerged about which political and structural information was available for extraction, which was only indirectly indicated, and which could not be identified because it was not reported. These recurring observations formed an important basis for the subsequent synthesis (Tables 1 and 2).

**Table 1:** Overview of the included systematic and scoping reviews

Study	Type	Policy Domain	Key Focus	Relevance to This Study
Volf et al. (2022) [62]	Systematic mixed studies review	Physical Activity and Sports policies	Impact on physical activity and sport participation	Demonstrates policy effectiveness in promoting physical activity
Woods et al. (2021) [63]	Systematic review	School-based physical activity policies	Impact on physical activity outcomes	Provides evidence on policy effectiveness in educational settings
Chugh et al. (2023) [64]	Systematic review	Tobacco control policies	Global impact on smokeless tobacco use	Highlights the effectiveness of tobacco control policies
Forberger et al. (2022) [65]	Scoping review	Smokeless tobacco control policies	Factors reported in research studies	Determinants covered in research studies
Forberger et al. (2022) [66]	Scoping review	Sugar-sweetened beverage taxes	Implementation processes	Barriers and facilitators in policy implementation
Forberger et al. (2022) [67]	Scoping review	Public physical activity policies	Implementation processes	Barriers and facilitators in policy implementation
Lobczowska et al. (2022) [68]	Systematic review	Healthy nutrition & physical activity policies	Frameworks for implementation evaluation	Provides methodological guidance for structured evaluation

All reviews adhered to PRISMA and PRISMA-ScR guidelines and were pre-registered in PROSPERO and OSF (CRD42020191946, CRD42019133251 [69, 70]). All referenced articles are published [62–68]. To guide data extraction in the scoping reviews, we developed a predefined, theory-informed data extraction form drawing on public policy, policy process, and policy implementation research (Appendix 2). This extraction form was developed as part of the registered review protocol [69]. It served as an operational tool to systematically identify and document how the included studies described public policies and their implementation processes. Appendix 2 documents the predefined categories, their characteristics, and the literature that informed their development. The predefined extraction form included metadata on publication and the public policy, including jurisdiction, country of origin, policy field, responsible public body, dates of policy adoption, revision and termination, and policy aims. It further captured characteristics of the study and policy implementation, including the theoretical approach used, policy operationalisation, implementation structures, organisational arrangements, actors involved, decision-making processes, discretion, positions of actors, the role of target groups, evaluation, and policy outcomes. Information was classified as explicitly reported, implicitly indicated, or not reported. Importantly, “absence of reporting” was not interpreted as absence of the determinant itself since publications may not provide sufficient information to determine whether a determinant was genuinely absent or simply omitted. The lead author was involved in all review stages across the included body of work, contributing to methodological continuity in considering political and structural aspects of policy implementation. The underlying review protocols and publications report detailed methodological procedures for study selection, data extraction, duplicate procedures, reviewer roles, coding, and discrepancy resolution. The synthesis drew on accumulated findings, bringing together evidence from the underlying reviews and examining which policy-related dimensions were consistently documented, which were repeatedly underreported or only indirectly indicated, and which reporting problems recurred across policy domains and implementation contexts. We consolidated the categories in the predefined extraction form and the recurring reporting gaps identified across the underlying reviews into eight overarching reporting dimensions (Table 2, operationalisation Table 3).

**Table 2:** Mapping of predefined data-extraction categories (Appendix 2) and recurring reporting gaps to the eight proposed reporting dimensions

Dimension	Main Source in the Extraction Form	New Elements from Reporting Gaps	Status
1. Policy Objectives & Scope	Categories 6–13, 16 (policy metadata)	Official name, legal reference, amendments	Consolidation
2. Actors, Power, Competence	Category 17 (actors, personnel, positions)	Formal vs. informal power, competence mismatch	Consolidation + Expansion
3. Legal Status & Jurisdiction	Categories 6, 16, 17 (partially)	Bindingness, enforceability, legal hierarchy	Consolidation + Expansion
4. Policy Instruments	Not explicitly covered in the form	Instrument typology, instrument mix	New (from reporting gap + policy theory)
5. Implementation Strategies	Category 17 (structure, decision-making, discretion)	Coordination mechanisms, street-level adaptation	Consolidation + Expansion
6. Resources	Category 17 (resource allocation)	Government implementation costs, transaction costs	Consolidation + Expansion
7. Equity Impact	Categories 17, 20 (target group, outcomes)	Equity aims, marginalised groups, path dependence	New (from reporting gap + equity literature)
8. Policy Lifecycle	Categories 10–12, 19 (dates, evaluation)	Lifecycle phase, evidence-informed adaptation	Consolidation + Expansion

The resulting eight dimensions therefore did not one-to-one reproduce the original extraction categories. Rather, they consolidate and further specify the policy-related information captured in the extraction form, combined with recurring reporting gaps and methodological observations identified across the underlying research. Categories were grouped where they addressed complementary aspects of the same broader reporting requirement and retained separately where they represented a distinct dimension necessary for interpreting policy implementation. The final dimensions and their reporting specifications were subsequently contextualised and refined using insights from the case studies.

### Case studies

The case studies were purposively selected based on research projects (PEN, STOP, ASTRA) in which the authors were directly involved, ensuring access to rich, context-specific data on policy implementation. Cases were chosen to represent a range of policy domains, governance levels, and socio-political contexts, enabling triangulation and cross-case comparison. Each case by itself was informed by a theory-driven design and used mixed-methods data collection to capture both structural and political dimensions of implementation. Appendix 1 provides detailed descriptions of the case studies, including study design, sampling, data collection, and analysis. In this synthesis, the case studies were not used to generate an independent sampling frame of determinants, but to examine, contextualise, and refine the operationalisation and reporting categories derived from the review work. They also provided context for interpreting why specific information was important for implementation evaluation and how gaps in reporting could limit the interpretation of implementation processes and outcomes. The three cases cover the EU School Fruit Scheme [71], physical activity policy in Baden-Württemberg (Germany) [72], and tobacco control in Khyber Pakhtunkhwa (Pakistan) [73–75].

## Results

Drawing on the integrative synthesis of findings from the underlying reviews and case studies, eight overarching reporting dimensions for political and structural determinants were consolidated as particularly relevant for understanding and reporting health policy implementation: (1) policy objectives and scope, (2) actors, power, and competence, (3) legal status and jurisdiction, (4) policy instruments, (5) implementation strategies, (6) resources, (7) equity, and (8) policy lifecycle.

The eight dimensions were refined into a practical operationalisation that specifies the information what to report and explains its relevance for evaluating policy implementation (Table 3). The operationalisation is intended to support systematic consideration and reporting and to reduce uncertainty regarding whether relevant dimensions are absent from a policy context or simply not visible in the available evidence.

**Table 3:** Overview of the eight reporting dimensions: key policy determinants, their operationalisation, and an explanation of why the information is important

Dimension	What should be reported	Examples	Explanation (Why?)
(1) Policy Objectives and Scope	Official name, adoption/termination date, issuing institution, implementing institution, amendments, geographical scope, stated aims and goals	EU fruit and milk scheme: Commission Delegated Regulation (EU) 2017/40 of 3 November 2016 supplementing Regulation (EU) No 1308/2013 of the European Parliament and of the Council with regard to Union aid for the supply of fruit and vegetables, bananas and milk in educational establishments and amending Commission Delegated Regulation (EU) No 907/2014; In force: This act has been changed. Current consolidated version: 31/01/2025	Ensures clarity, transparency, and rigour; Helps identify the policy and its evolution; Clarifies governance and power structures; Identifies the aims and all goals of the policy
(2) Actors, Power, Competence	Actors involved (state/non-state, private person), their competencies and power	Ministry of Health (authority), NGOs (influence), street-level bureaucrats (administration)	Distinguishes between formal legislative or enforcement power and informal influence; Explains actor roles in outcomes; Refers to the institutional and policy-field specificity of an actor’s mandate; Reveals power dynamics, coordination needs, and legitimacy of implementation
(3) Legal Status & Jurisdiction	Jurisdictional level (EU, national, local), bindingness, enforceability, and clarity of legal instruments	EU regulation (directly binding in all Member States upon entry into force, requiring no transposition), EU directive (binding on results, but Member States must transpose them into national law within a deadline), national regulation (enforceable), campaign (non-binding)	Clarifies scope and enforceability; Different governance levels have distinct powers and influence; Clarifies legislative competence; Legal quality affects enforcement capacity; Determines implementation authority, compliance, and sustainability
(4) Policy Instruments	Type and mix of instruments used (regulatory, economic, communicative, behavioural)	Regulatory (bans, mandates), economic (taxes, subsidies), communicative (campaigns), behavioural, instrument mix per implementation level; Examples: Tax on sugary drinks, school fruit provision, public awareness campaigns	Instrument choice reflects political, social, and institutional context; Affects behaviour, acceptance, enforceability and impact; Influences acceptability, feasibility, and behavioural impact
(5) Implementation Strategy	Organisational approach: vertical, horizontal, hybrid; coordination mechanisms; centralised vs. decentralised decision-making, coordination mechanisms, adaptation space, de-implementation; Cross-sector cooperation and stakeholder involvement	Top-down, bottom-up, mixed approach; Cross-sector collaboration, integration across levels; Local adaptation Inter-ministerial groups, school involvement, citizen participation, and tools used for facilitation	Impacts adoption and sustainability; Is influenced by administrative culture and organisational patterns; Shapes implementation fidelity, flexibility, and stakeholder engagement Enhances legitimacy, acceptance, and policy quality, but may shift power balances; Tool choice helps to identify barriers and facilitators; Report should also capture access pathways, position development, and adaptation (closely related to 2)
(6) Resources	Financial, human, infrastructure and material support for frontline and government actors; Implementation and implementation process costs (training, monitoring)	Budgets, staffing levels, training programmes, infrastructure, and IT systems, administrative burden	Resources are prerequisites; Overlooking government implementation costs leads to gaps and inefficiencies; Determines the feasibility and sustainability of implementation
(7) Equity Impact	Whether the policy aims to reduce disparities and whether marginalised and/or minoritised groups are involved; Equity is incorporated into the policy	Targeted outreach to low-income communities, inclusion of marginalised voices (closely related to 2, 4, 5)	Ensures policies do not increase inequities; Captures intended and unintended consequences, Allows monitoring of the impacts of historical policy trajectories
(8) Policy Lifecycle	Phase of the policy cycle (design, implementation, evaluation, termination)	Ongoing implementation, policy review, or sunset clause	Contextualises findings and informs future policy cycles; Positions policy implementation evaluation as an integral part of the policy cycle and policy adaptation

### Policy objectives and scope

Papers often lack basic information about the object and type of public policy under evaluation. Therefore, before commencing evaluation, we advocate for clearly naming and specifying the public policy assessed. This should include details such as the year of adoption, any subsequent amendments, the policy’s duration (if applicable), the institution responsible for its implementation, and the jurisdiction to which the policy applies (e.g., city, county, state, or national). This foundational information helps classify the policy as a governmental action, ascertain enforcement power and delineate the scope of the public policy under evaluation. By establishing this groundwork, evaluations can be clearer and more effective, ensuring the real-world context in which the policy is enacted is accurately captured. For example, the EU School Fruit and Milk Scheme should be reported with its full legal reference: Commission Delegated Regulation (EU) 2017/40 of 3 November 2016, including the date it entered into force and any subsequent amendments (e.g., consolidated version as of 31 January 2025).

### Actors, power, and competence

Effective policy implementation across nutrition, physical activity promotion, and tobacco control requires active involvement from diverse stakeholders, including governmental, quasi-governmental, and non-governmental organisations, private stakeholders, and lobby and advocacy groups. Because power and competence asymmetries shape collaboration, evaluations should document actors’ roles and positions. Power can be defined as either formal legal authority or informal influence. State or quasi-state actors, institutions, or organisations acting on behalf of governmental bodies often hold different legal and normative power than community-based actors [76, 77]. Formal legal authority, such as that held by ministries or regulatory agencies, does not always equate to practical influence. Conversely, community-based organisations or civil society actors may exert significant informal or quasi-normative power, particularly in contexts where state capacity is limited or fragmented [76, 77]. Especially where governmental actors are absent, it is important to report on the actors and their positions, as they may set norms. Competence is defined as the extent to which a policy measure falls within an actor’s core mandate and traditional policy domain. It reflects how well implementation tasks align with the actor’s institutional responsibilities, legal authority, and established administrative expertise. Evaluations should therefore systematically document the stakeholders involved, including their legal status (e.g., governmental institution, non-governmental organisation, or private individual), their field of activity, and the broader policy context. In addition, reporting should capture their formal authority, informal influence, implementation responsibilities, and the extent to which the policy task aligns with each actor’s institutional mandate. This information helps identify actors’ roles across different contexts and implementation stages, including whether they function as decision-makers, implementers, intermediaries, advocates, or veto players [48, 78].

Such reporting clarifies how implementation is organised, which actors can make or enforce decisions, which shape norms and acceptance, where responsibilities are fragmented, and where gaps in competence or legitimacy may hinder implementation. It also helps evaluators understand how actor configurations shape outcomes. For example, whether weak coordination between responsible agencies delays delivery, whether actors with strong informal influence facilitate uptake, or whether implementation tasks are assigned to organisations without a clear mandate or sufficient capacity.

For example, in the EU School Fruit Scheme case, implementation involved actors across multiple governance levels. Actors include the European Commission as the issuing institution with formal authority, and national ministries of agriculture as implementing institutions with formal competence but limited coordination knowledge within the school system. Local schools under the authority of the national ministries of education are the frontline implementers, with high discretion but limited influence on policy design. Power asymmetries between these actors, particularly the mismatch between ministerial responsibility and school-level implementation capacity, were identified as a key implementation barrier [71].

This approach supports a nuanced analysis of power dynamics, coordination needs, and legitimacy gaps. More specifically, it can inform implementation strategy design by identifying where stronger intersectoral coordination, clearer role definitions, stakeholder engagement, or capacity-building measures are needed. It also reveals how actors navigate institutional constraints, adapt to local realities, and ultimately shape implementation outcomes, not merely as passive implementers, but as active agents in the policy process. For policy implementation evaluation, this information matters because it supports a more context-sensitive interpretation of success or failure and helps distinguish whether implementation problems stem from policy design, actor constellations, governance arrangements and mandate mismatches.

### Legal status of the policy

A policy’s legal status (binding law, non-binding recommendation, voluntary programme) determines its enforceability, scope, and implementation authority. This includes national legislation, local regulations, international agreements (e.g., the WHO Framework Convention on Tobacco Control), and supranational instruments such as EU directives. They allocate responsibilities, set standards, and establish authority and enforcement mechanisms. Differentiating between laws, proposals, and campaigns helps assess the degree of coercion involved and the potential impact on behaviour. While laws mandate implementation, other instruments, such as guidelines or recommendations, lack legal enforcement power but can still be implemented effectively through strategic coordination [79, 80]. Coercion describes the degree of compulsion. Binding mandates backed by enforceable sanctions involve greater coercion than non-binding guidelines, which rely on voluntary compliance. This distinction also highlights how instruments shape accountability and power relations [81].

Within national legal systems, laws enacted by parliament carry the full force of law and obligate state actors to implement them. Regulations and administrative directives, issued by executive bodies, derive their authority from legislation and can operationalise it by translating statutory goals into rules, procedures, standards, and administrative responsibilities. The parent law may therefore limit their scope and enforcement options. This means that regulations and administrative directives are not only implementation instruments in their own right, but also part of the legal chain through which legislation becomes actionable in practice. In contrast, non-binding instruments, such as policy guidelines, action plans, or public awareness campaigns, lack legal enforceability but can still influence behaviour through normative pressure, stakeholder engagement, or strategic coordination. Political strategies and programmes typically set priorities without statutory obligations, relying on voluntary compliance or subsequent regulation. Depending on the legal system, delegated instruments may also be subject to requirements such as administrative review, consultation, publication, or judicial oversight.

Reporting on these relationships is therefore important for implementation evaluation, as it helps distinguish whether implementation is governed directly by primary legislation or mediated through secondary or administrative acts, and whether delays, inconsistencies, or constraints arise from the design of these delegated instruments rather than from the law itself.

At the supranational level, the European Union’s legal architecture exemplifies the complexity of multi-level governance. While EU regulations are directly applicable and binding in all member states, EU directives must be transposed into national law, creating variability in implementation because of differences in institutional capacity, political will, and administrative culture [82, 83]. Directives’ flexibility allows contextual adaptation but may also lead to inconsistent implementation and uneven enforcement.

These points are addressed by distinguishing policy design specifications (Category 3) from actual implementation and adaptation strategies (Categories 5 and 6). This separates design intent from adaptation and execution, and clarifies whether uneven implementation stems from deliberate policy flexibility, suboptimal adaptation strategies, or implementation failures, facilitating targeted improvements across the policy-implementation continuum.

WHO policies and guidelines, as an example for an international organisation, while influential and internationally respected, generally carry normative, not legal, power [84, 85]. They provide crucial normative frameworks and technical guidance without legally binding force, relying instead on member states’ voluntary adoption and contextual adaptation. Most WHO policies and technical guidance derive their authority primarily from moral and scientific legitimacy rather than formal legal status, distinguishing them from national legislation and EU law. However, the WHO also issues legally binding instruments, notably the International Health Regulations (2005) and the WHO Framework Convention on Tobacco Control (WHO FCTC). Whereas the WHO FCTC is a treaty that requires ratification and subsequent transposition into domestic law [86], the IHR (2005) constitute binding regulations for WHO Member States (subject to the IHR opt-out provisions) and requires the development and maintenance of core public health capacities. These distinctions matter for implementation evaluation because legal bindingness alone does not ensure effective implementation, which may be undermined by weak enforcement, limited resources, or misalignment with local contexts [87]. Accordingly, robust assessment should combine contextual analysis with process evaluation to examine how international legal frameworks are translated into practice and embedded within existing legal and administrative structures [76].

### Policy instruments

In addition to identifying the policy’s legal status, it is important to specify the instruments the policy employs (Table 4).

**Table 4:** Typology of policy instruments [87–90]

Policy Instruments	Description	Types of instruments
Substantive Tools: a) Communicative (Information)	Attempts to influence people’s behaviour through information (good vs. bad; allowed vs. not allowed; should be done vs. should not be done (including normative dimension))	Mediated transmission: TV, radio, film, newspaper, printed matter, labels, posters Interpersonal transmission: direct personal advice, classroom or on-site education, workshops, conferences, demonstrations, government examples, exhibitions, investigation and publicity
b) Regulative	Governmental units take measures to influence behaviour through formulated rules (with negative consequences or without (lex imperfecta))	Unconditional (absolute) prohibitions Conditional prohibitions: with exemptions, with permissions (concessions, permits, licenses, authorisations), with obligations to notify
c) Economic	Handing out or taking away material resources (cash or in-kind)	Incentives (affirmative, promoting, encouraging) e.g., cash transfer & grants, subsidies, reduced interest loans Disincentives (negative, restraining, discouraging) e.g., taxes, charges, fees, customs duties, tariffs, taxes, charges, fees, customs duties, tariffs In-kind (incentives) e.g., government provision, private provision, vouchers
Procedural tools	Mechanisms or strategies employed by governments to facilitate the policy design, implementation, and evaluation processes, with a focus on how decisions are made and actions are carried out, can enhance the effectiveness, transparency, and accountability of the policymaking process, and can be digital.	Deliberative process design: e.g., voting regulations, stakeholder engagement, e.g., by changing or altering networks or creating advisory committees, types of stakeholder involvement (citizen science, advisory boards, public consultations), audits, evaluations, monitoring, data access, transparency mechanisms, use of digital tools for the engagement process
An approach to designing the instrument		
Behavioural Public Policy	Instruments informed by insights derived from the behavioural and social sciences include decision-making, psychology, cognitive science, neuroscience, economics, and organisational and group behaviour	- Educational nudges (e.g., labels, warnings) - Architectural nudges (e.g., defaults, TIPME (typology of interventions in proximal physical micro-environments) [91]) - Sludge audits (to simplify processes and access) - Use of behavioural insights to improve existing policies (such as taxes and regulations)

Policy instruments shape behaviour, compliance, and institutional action through different mechanisms. Communicative instruments, such as public information campaigns, media, and direct education, aim to inform and normatively influence behaviour by shaping public attitudes and choices through persuasion and awareness [66, 92]. Regulative instruments impose formal rules, often with sanctions, directly mandating or prohibiting actions. Economic instruments provide incentives or disincentives that influence resource allocation and individual or organisational behaviours through financial mechanisms [65, 75]. Behavioural public policy is an approach to designing instruments based on insights from the behavioural sciences, using nudges and architectural designs to guide decisions subtly [93]. A policy mix, the strategic combination of multiple instruments, is often more effective than single measures. For example, in tobacco control, a comprehensive package may include indoor smoking bans (regulative), higher tobacco taxes (economic), and public awareness campaigns (communicative) to reduce demand and improve public health [64, 74, 75]. In addition to substantive tools, governments can employ procedural tools, mechanisms that structure the processes and methods through which policies are implemented and enforced. These tools focus on the decision-making procedures with deliberative mechanisms (e.g., stakeholder consultations, advisory committees, public hearings) that enhance legitimacy and inclusion, employing monitoring, evaluation, and audit systems to ensure accountability and transparency and using digital tools (e.g., data platforms, e-service delivery) to improve efficiency and accessibility [94]. These tools can affect acceptance and, in turn, implementation, shaping implementation outcomes. Transparent reporting of instrument choice and mix is essential to assess how policies pursue their objectives, explain implementation outcomes, identify synergies or conflicts, and understand political acceptability and feasibility.

### Implementation strategies

**Fig. 1:**
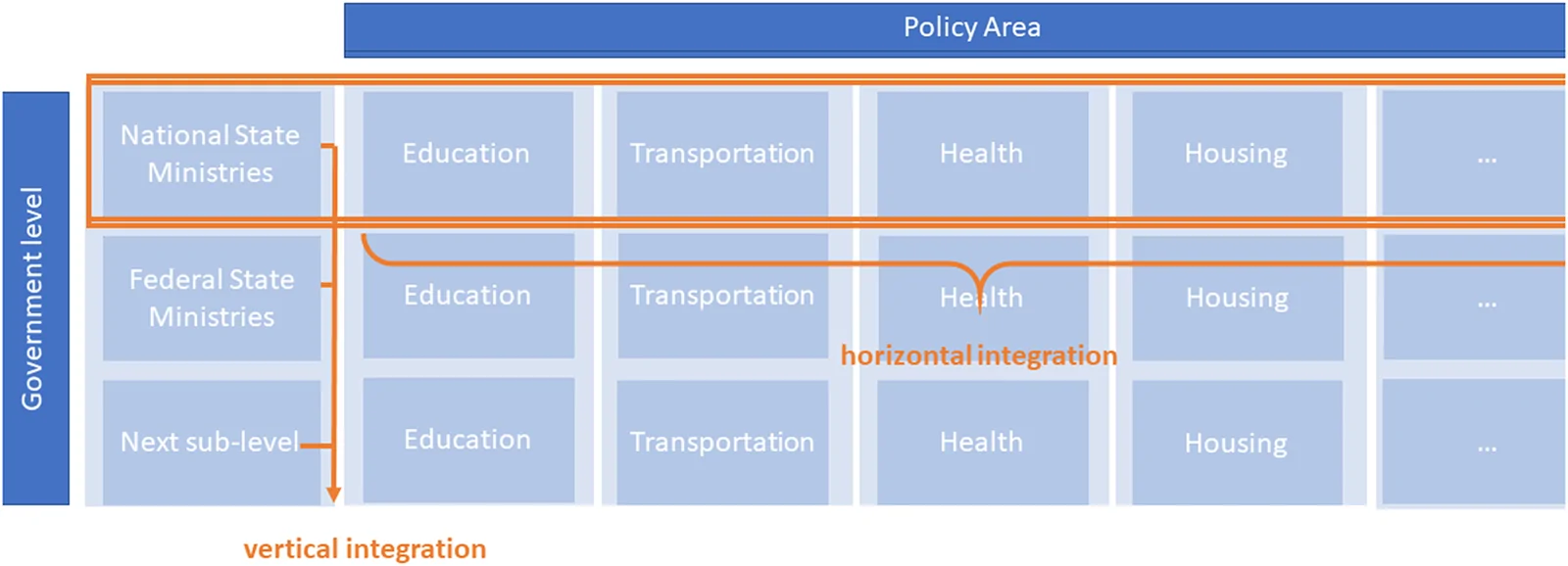
Horizontal and vertical integration adapted from Kettner et al. 2012 [97]

Implementation strategies encompass vertical (top-down and bottom-up) and horizontal (cross-sectoral, collaborative) approaches (Fig. 1). Effective implementation often requires coordination and cooperation among diverse stakeholders, such as educational authorities, health agencies, and food suppliers. Moreover, administrative traditions and organisational cultures significantly shape implementation dynamics within national contexts [95, 96].

For example, direct provision programmes, such as the EU School Fruit Scheme, involve complex logistical coordination and quality control across multiple ministries responsible for consumer protection, agriculture, education, and transport. Each ministry and authority operates under different administrative procedures and cycles. Although the scheme is designed at the EU level, its success depends on horizontal integration across policy domains, particularly between agricultural and educational planning. However, misaligned administrative schedules, with agriculture operating on an annual cycle and education following the academic year, have been identified as a determinant of implementation delays [71]. Similar challenges have also been observed in the implementation of physical activity policies in school settings.

In physical activity promotion, federal and state regulations are often combined with community-led initiatives. These efforts rely on partnerships between local governments and non-governmental community organisations and agencies to ensure contextual relevance and sustainability [67]. However, implementation of physical activity policies in school settings showed predominant vertical integration and limited cross-sector cooperation [67].

This tension between vertical and horizontal integration is critical. While horizontal collaboration enhances coordination, efficiency, and policy coherence across sectors, vertical alignment between frontline implementers and decision-makers is essential for contextual adaptation, empowerment, and legitimacy [98, 99]. As Lilly et al. (2023) note, while horizontal coordination is often well-established, vertical collaboration remains underdeveloped, particularly between street-level bureaucrats and central policymakers [100].

Moreover, implementation is not merely policy execution. When policies reach the frontline, the formal policy framework often recedes into the background, giving way to practical implementation practices. Street-level bureaucrats exercise significant discretion in adapting policies to local realities, client needs, and institutional constraints [101, 102]. A recent systematic review shows that these actors often develop coping mechanisms, such as purposeful client involvement or informal negotiation, that can reshape policy outcomes in ways designers did not anticipate [103]. Reporting implementation strategies requires distinguishing formal design from actual execution and adaptation. This distinction matters especially in public policy implementation. FRAME-IS and LISTS can provide established typologies for documenting planned versus emergent adaptations and strategy fidelity, though policy contexts require extensions to capture, e.g., multi-level governance dynamics and street-level bureaucrat discretion [104, 105].

Therefore, implementation strategies must be reported not only in terms of formal design (top-down vs. bottom-up) but also in terms of actual coordination mechanisms, adaptation space, and the role of frontline actors. Without this insight, evaluations risk misrepresenting the true nature of implementation and missing key leverage points for improvement.

### Resources

Studies often identify financial, human, and material resources as missing or unreported, despite their importance for policy implementation. Both frontline implementers and the public administrations responsible for execution require resources, including infrastructure, staff training, public awareness, and enforcement. However, implementation costs borne by governmental actors themselves are often overlooked. It is often assumed, explicitly or implicitly, that these resources are already in place and, therefore, do not warrant further consideration. Yet baseline administrative capacity and infrastructure, including personnel, IT systems and their maintenance, training, manuals, and materials, should also be included in total implementation costs. These costs may include strategy development, integrating a regulation into an existing system or establishing a new one, and ongoing administration [106]. For example, the OECD (2007) quantifies policy-related transaction costs (implementation costs) across agricultural policies, distinguishing compliance costs (foregone revenue, abatement) from administrative transaction costs (information gathering, enactment, monitoring, prosecution) [107]. Further, costs are influenced by institutional arrangements, including the clarity of objectives (Category 1), implementation strategies and coordination (Category 5), and the number of participating actors and agencies (Category 2) [108–111]. However, for the implementation of population-based policies, these costs are rarely specified and reported. As a result, policy planning, budgeting, and implementation design often do not adequately account for these costs. This contributes to a persistent underestimation of the resources required for implementation and, in turn, to the resource constraints that frequently undermine policy. This omission can lead to underestimating required resources and mismatches between policy demands and administrative capacity and can hinder policy performance, resulting in inefficiencies and reduced sustainability [112]. It is therefore both a reporting gap and a governance challenge. Administrations need resources to coordinate, enforce, and sustain policies, while the associated transaction and administrative costs must be anticipated and budgeted. Resources are a core determinant of implementation feasibility, quality, and sustainability. Fernández-i-Marín et al. (2024) and Forberger et al. (2022) demonstrate that public administrations are often overburdened when tasked with additional policies without a corresponding increase in resources. Thus, successful policy implementation depends on aligning policy expansion with available administrative capacities [66, 98].

### Equity considerations

Equity is a cross-cutting dimension relevant to policy concepts and all stages of implementation, although it is treated here as a separate category for analytical purposes [54]. Equity can be embedded at the policy formulation stage, particularly when policies explicitly aim to address the needs of marginalised and/or minoritised groups [113–115]. Marginalised groups experience systematic exclusion and limited influence in social, economic, and political systems. Marginalisation refers more broadly to being pushed to the margins of society and its institutions. Minoritised groups are those whose minority status is produced and maintained through power structures and institutional practices. Minoritisation emphasises how certain groups are actively constructed as ‘minorities’ through discriminatory structures and practices, even if they are not numerically small. Both terms describe populations who are systematically disadvantaged or excluded from social, economic, and political systems, often as a result of intersecting identities such as race/ethnicity, socioeconomic status, disability, gender, or sexual orientation. Although these groups often overlap, not all marginalised groups are minoritised, and not all minoritised groups experience the same forms of structural disadvantage. However, they are not interchangeable. Groups’ specific barriers and needs vary by context and intersecting identities [114, 116]. Nevertheless, equity objectives are most often realised during operationalisation and implementation, where systemic barriers and contextual factors frequently hinder equitable access and outcomes [46, 117, 118]. To effectively embed equity across policy concepts and implementation stages, it is essential to move beyond individual-level interventions and the explicit inclusion of marginalised groups, and to account for path dependence, anchoring effects, and historical policy impacts [119]. Such institutional lock-ins can lead even well-intentioned, equity-neutral policies to reinforce disparities. Recognising and counteracting these legacies is essential to reducing systemic barriers and promoting equitable access [6–8].

For example, frameworks such as Equity in Implementation Research (EquIR) or the Health Equity Implementation Framework provide theoretical and practical guidance for embedding equity throughout the implementation process by recognising differential needs, power dynamics, and social determinants of health [68, 120]. Beyond implementation science, additional theoretical perspectives, such as critical race theory, feminist theory, postcolonial theory, and structural theories of health inequalities, offer important lenses for analysing power structures, systemic inequities, and the social distribution of benefits and burdens in relation to legal frameworks addressing structural racism in policy implementation evaluation [113]. This entails participatory approaches that meaningfully engage affected communities, iterative adaptation to local contexts, and the establishment of accountability mechanisms sensitive to equity-related outcomes [117, 121]. These considerations align particularly closely with Categories 2 (actors), 4 (tools), and 5 (implementation strategies).

Failure to embed equity systematically risks perpetuating or exacerbating existing disparities, thereby undermining the legitimacy and effectiveness of public policies. Consequently, advancing equity-centred policy implementation requires both explicit theoretical framing of equity within frameworks and methodological innovations that capture the complexity of social inequities in real-world settings, integrating these aspects into implementation evaluation [54].

### Policy lifecycle

Although evaluation may focus on implementation, a policy’s lifecycle stage-design, implementation, evaluation, or termination-shapes implementation dynamics, stakeholder engagement, and evaluation priorities, yet is often overlooked. Policies are not static. They evolve through distinct stages, each with unique challenges and opportunities. During the design phase, policymakers define goals, target populations, implementation pathways, and institutional structures. In the implementation phase, policies are operationalised, resources are mobilised, and frontline actors begin to enact them. The evaluation phase allows for monitoring progress, identifying barriers, and assessing impact. Finally, the termination or renewal phase involves sunset clauses, policy review, and systematic policy adaptation. Rather than assuming that policies themselves are fully evidence-based, policy adaptation should be understood as an evidence-informed process [122]. Adaptation decisions may draw on scientific evidence, including evaluations, systematic reviews, and other empirical findings, while also considering political feasibility, institutional capacity, stakeholder interests, and contextual constraints. This balance is a core reason why policy implementation scholarship commonly refers to evidence-informed rather than evidence-based policymaking [38, 131].

Crucially, the phases do not operate in isolation [123]. Structures and decisions that evolve or are deliberately developed and built during the design phase have lasting effects on subsequent stages [124]. For example, strong institutional frameworks established early that incorporate all stakeholders and their perspectives support a long-term vision and the governance and procedural structures that can facilitate later implementation. Clear, transparent funding mechanisms early on signal a government’s stance and support implementation. This can enhance stability and protect policy integrity by shielding it from external pressures and short-term political shifts, ensuring continuity, and supporting the achievement of long-term objectives [125, 126].

Yet, under specific circumstances, once established and embedded, such structures can also hinder adaptability. These structures may resist necessary changes, preventing the integration of new evidence, emerging needs, or stakeholder feedback. This can lead to implementation stagnation, reduced responsiveness, and inequitable outcomes, particularly in dynamic or complex environments. Conversely, a flexible implementation and evaluation design that accounts for these structures is better equipped to anticipate change and incorporate feedback loops [127]. For instance, policies with built-in review mechanisms or adaptive implementation pathways are better equipped to respond to emerging challenges and incorporate lessons from evaluation into ongoing implementation [32, 128–130].

Reporting how earlier lifecycle stages shape current implementation helps contextualise findings, compare policies, and support iterative improvement [55].

## Synergising implementation and policy research

This paper illustrates how the identified political and structural determinants and their operationalisation can complement established implementation science frameworks, such as CFIR and CICI. While these frameworks address organisational, political, legal, and other contextual influences, policy-specific political and structural determinants are not always operationalised in enough detail to guide systematic consideration and reporting. The proposed operationalisation offers additional guidance for making these policy-specific dimensions more explicit. Rather than replacing or formally modifying established frameworks, the eight dimensions provide additional guidance for identifying, examining, and reporting political and structural influences on policy implementation. The dimensions may support a more explicit examination of policy implementation as a process shaped by policy design, governance structures, actors, legal arrangements, and institutional context.

This operationalisation can serve as a structured guide for incorporating the identified determinants into implementation evaluations. Beginning with precise policy identification, the process may involve mapping actors and their positions, analysing legal frameworks and instruments, detailing policy-specific implementation strategies, assessing resources, and systematically reporting the relevant dimensions (Table 3). For each determinant, the operationalisation provides specific information to consider and report, which may support more transparent and context-sensitive data collection and analysis. For example, identifying and mapping involved actors can be conceptually related to CFIR’s domains “Inner Setting” and “Characteristics of Individuals”, extending attention to formal authority, informal influence, competence, and power relations. Correspondingly, the analysis of legal frameworks and policy instruments can be considered alongside CFIR’s “Outer Setting” domain, adding policy-specific detail on legal status, jurisdiction, enforceability, and the mix of policy instruments. Implementation strategies can also be linked to the CFIR “Process” domain, drawing additional attention to vertical and horizontal coordination and adaptation space. The assessment of resources may complement CFIR’s consideration of available resources and capacity by specifying policy-related resource requirements and implementation costs. From the perspective of the CICI framework, these steps resonate with its multi-level approach, which incorporates intervention, context and implementation dimensions. For both frameworks, the proposed operationalisations are conceptual points of connection and not a formal extension. Not all determinants will be equally relevant to every policy or evaluation question. Where a determinant is not applicable or is considered outside the scope of a specific evaluation, this should be made explicit in the reporting. Such transparency may help distinguish between dimensions that are genuinely irrelevant to a particular policy context, dimensions that were outside the scope of the evaluation, and dimensions for which relevant information was not examined or reported.

## Discussion

The proposed operationalisation responds to a gap identified in the reviewed literature by providing systematic guidance for considering and reporting political and structural determinants of health policy implementation, as acknowledged in frameworks such as CFIR and CICI but under-specified in practice. While these frameworks recognise organisational, political, legal, and other contextual influences on implementation, policy-specific and structural determinants may not always be operationalised in sufficient detail to guide systematic reporting in health policy implementation evaluations.

For instance, legal characteristics can be described more specifically in terms of bindingness, enforceability, and clarity rather than being reported only as part of a broad policy context. These characteristics may influence implementation fidelity and compliance. Similarly, policy instruments are not treated as generic “tools”. Rather, they can be differentiated by type and mix, which may support more detailed examination of how different instruments shape implementation dynamics. Furthermore, implementation strategies are assessed not only in terms of top-down versus bottom-up approaches, but also in terms of their coordination mechanisms and the degree of adaptation space afforded to implementers. These factors are particularly relevant for understanding sustainability and contextual responsiveness.

This operationalisation is intended to support more consistent reporting across studies and contexts and may thereby facilitate comparison. Establishing clear, transparent criteria for reporting policy implementation processes and relevant determinants may contribute to transparency and provide a basis for assessing the reproducibility and credibility of findings in future research. The proposed structure may also support evidence-informed decision-making. Moreover, the structured approach may help researchers identify potential leverage points within implementation pathways. The operationalisation does not establish which points are most influential or whether targeting them would produce greater impact. This may inform future work on refining more adaptive and context-sensitive implementation strategies.

Table 3 can be used to specify implementation frameworks. Rather than replacing existing frameworks, the operationalisation can be used alongside them to make policy-specific dimensions more explicit and thereby enrich their analytical depth. The operationalisation draws on and organises concepts from established frameworks and policy research. Bullock et al.‘s (2021) actor typology conceptualisation (context, innovation itself, enabler/barrier, implementation outcome) is operationalised in, e.g., Category 2 “Actors, Power, Competence,” which systematically documents state versus non-state actors’ formal legal authority, informal influence, and specific implementation roles across the policy process. Legal and jurisdictional arrangements describe the governance structures and formal rules within which actors operate, while implementation strategies capture coordination mechanisms, administrative arrangements, and cross-sector collaboration [48]. Similarly, Crable et al.‘s (2022) six policy dimensions for strengthening the application of the EPIS framework can be related to the proposed dimensions. Policy function and form are addressed through policy objectives and scope and legal status and jurisdiction. Nonlinear implementation phases are captured through the policy lifecycle. Stakeholder roles are considered within actors, power, and competence. Contextual adaptation is addressed through implementation strategies, and coordination mechanisms can support the examination of bridging factors [41].

McLoughlin et al.‘s (2024) equity-informed measurement recommendations, which require policy-specific disparity-reduction aims and marginalised-population inclusion throughout implementation, are reflected in Category 7, “Equity Impact,” which supports the explicit consideration of whether policies address structural inequities, incorporate the perspectives of marginalised populations, and consider the distribution of policy effects and historical inequities [46]. This synthesis connects concepts from implementation science and public policy research by translating theoretical insights into concrete reporting dimensions informed by recurring observations across reviews and diverse policy implementation contexts. The operationalisation complements existing implementation science structures, such as CFIR and CICI, and is potentially adaptable to different policy domains and governance contexts. However, its transferability requires further testing. This approach helps manage expectations more realistically and cautions against overly technical or deterministic views of implementation, instead acknowledging the inevitable role of political negotiation, competing interests, and institutional constraints in shaping outcomes [38, 131].

Despite these advances, evaluating policy implementation remains inherently complex due to multiple actors, overlapping behavioural domains, and the interplay of formal structures and informal practices. Current research is limited by language and database biases, inconsistent use of comprehensive frameworks, a disproportionate focus on high-income countries, ambiguous policy definitions, and insufficient attention to socio-cultural contexts. Methodological constraints, including fragmented data, low response rates, and limited longitudinal evidence, further hinder assessment of long-term implementation effects.

To address these challenges, future research should prioritise standardised data collection and reporting, greater geographical and sociocultural inclusivity, clearer policy definitions, and systematic integration of sociocultural analysis into evaluation designs.

This paper contributes to more rigorous reporting of political and structural determinants by making both available and missing information explicit. Even when questions cannot be answered, asking them distinguishes between information that was not examined, not reported, or deemed not applicable. These are distinctions that are often overlooked in current practice. This approach may enhance transparency and reporting consistency, although its contribution to the evidentiary basis of implementation research requires further assessment. The operationalisation provides a structured approach to linking theoretical frameworks with empirical reporting and, by systematically incorporating political and structural context, may support more context-sensitive, transparent, and equitable assessments of policy implementation.

A potential limitation of this work is that the case studies were drawn from projects in which the authors were directly involved, potentially introducing confirmation bias in the interpretation and selection of evidence. We were aware of this possibility and addressed it by grounding the core structure of the proposed dimensions in a literature-based body of work on implementation determinants and using this as a priori guidance for synthesising and interpreting evidence, rather than allowing the case study material to redefine the framework post hoc. We further strengthened this approach by drawing on multiple projects across different policy domains and contexts, and by systematically comparing findings across the reviews and case studies. Nevertheless, the purposive and project-based nature of case selection means that the findings should be interpreted as analytically informed rather than statistically representative. Future research would benefit from additional external validation, for example, through expert panels, facilitated stakeholder discussions, or Delphi-based procedures, to further refine and strengthen the proposed operationalisation. Further, this paper does not provide detailed guidance on methodological approaches (e.g., mixed methods design, stakeholder mapping) needed to collect the information specified in the operationalisation. Moreover, it does not address how to weigh or prioritise dimensions when trade-offs must be made, nor does it specify how to analyse interactions between dimensions. Future work should develop practical guidance on data collection methods, analytic strategies, and reporting formats that support the operationalisation’s integration into policy evaluation practice. Additionally, the paper focuses on nutrition, physical activity, and tobacco control to systematically identify reporting gaps in political/structural determinants across these domains. These policy domains revealed recurring reporting gaps that may also be relevant to other areas of public policy implementation. While they provided valuable cross-case insights, they do not represent the full spectrum of public health policies, governance structures, or implementation settings. For instance, health system policies, environmental health regulations, or policies in low-income or conflict-affected settings may involve additional political or structural determinants not fully captured by the present operationalisation. Further testing and potential adaptation of the operationalisation across a broader range of contexts is therefore necessary.

## Conclusion

Evaluating the implementation of health-promoting policies requires robust, systematic approaches that integrate theoretical insights, contextual considerations, and methodological rigour. The suggested operationalisation provides more detail to support the consideration and reporting of key political and structural determinants, potentially contributing to greater transparency and interpretability in policy implementation evaluations.

Transparency and consistency in evaluation may help clarify which factors are reported as relevant to implementation and may support feedback for policy refinement. Tailored, adaptive evaluation approaches that explicitly acknowledge political context and power dynamics can support a more nuanced understanding of implementation processes and their contextual influences.

The study underscores the value of integrating established implementation frameworks with insights from policy science to identify and operationalise key determinants of policy implementation. By grounding the policy-related contextual, structural, and actor dimensions of implementation in concepts from public policy research, this approach offers a structured set of prompts for considering and reporting information that may be relevant to interpreting implementation outcomes.

Improved reporting of policy implementation processes and outcomes may improve the interpretability of findings and provide a basis for feedback into policy and evaluation systems, thereby potentially supporting evidence-informed decision-making. This aligns with Oh et al.’s (2021) concept of a Learning Health Policy System, which advocates for iterative, dynamic evaluation processes that enable policies to be adapted in real time as they are implemented [32]. Such an approach underscores the importance of continuous stakeholder engagement, ongoing data collection, and responsive evaluation to navigate the complexities of policy implementation.

Policy implementation evaluation may benefit from being continuous, dynamic, and embedded within policy cycles, particularly where contexts and implementation challenges evolve. Mechanisms that enable timely communication, transparent reporting, and responsive policy adjustment are pivotal to translating evaluation insights into meaningful health improvements and to linking implementation science to methods from systems science [132].

Yet, to realise these advances, further work is needed to test and refine systematic operationalisation and structured reporting. This paper, therefore, offers a preliminary conceptual and practical resource for further development and testing.

## Supplementary Information

Below is the link to the electronic supplementary material.

Supplementary Material 1

Supplementary Material 2

## Data Availability

No datasets were generated or analysed during the current study.

## Abbreviations

CICI: Context and implementation of complex interventions (CICI) framework
CFIR: Consolidated framework for implementation research
