# Supplementary material for: Bridging policy and implementation science: operationalising political and structural determinants for health policy implementation evaluation
Source: Implement Sci Commun. 2026 Oct 11;7:202. doi: 10.1186/s43058-026-01137-0 (PMC13678997; doi:10.1186/s43058-026-01137-0)
Supplement: Supplementary file 1 [file 43058_2026_1137_MOESM1_ESM.docx]

## Appendix 1 : Overview of included case studies

**Table 1: Overview of Case Studies**

| **Case study** | **Short overview** |
| --- | --- |
| **Implementation of the EU School Fruit Scheme across ten EU countries** (1) | A qualitative study explored determinants affecting the implementation of the EU School Fruit Scheme across ten EU countries, employing the Consolidated Framework for Implementation Research (CFIR) to guide data collection and analysis. **Primary data collection was conducted using a qualitative, exploratory design. Semi-structured interviews were conducted with 29 representatives from 10 countries, primarily from ministries of agriculture, health, and education or related institutions responsible for implementing the Scheme. Recruitment used key informants and snowball sampling to ensure diverse perspectives. Interviews held online from June 2020 to June 2021 were one-on-one or with more than one representative. Consent was obtained, and interviews were recorded. The data analysis involved transcription, translation, and importation into NVivo 12 Pro. Based on Braun and Clarke’s approach, an inductive thematic analysis was combined with a framework analysis. The CFIR constructs were used to map inductive themes, ensuring a comprehensive exploration of barriers and facilitators. A diverse team of authors with expertise in nutrition, epidemiology, implementation science, political science, and psychology contributed to the analysis process.** Content was identified in relation to 19 out of 26 constructs of the CFIR. Some example constructs include adaptability, external policy and incentives, networks and communications, knowledge and beliefs, and execution. Flexibility in the design and implementation of the scheme, along with newly established cooperation between implementing ministries, acts as a facilitator. However, the timing of the yearly budget allocation presents a barrier. Taking EU funding for granted may serve as a potential disincentive to improvement, though it facilitates sustainability. Despite agreement on the scheme's overall goals, there is ambiguity about the perceived primary goal, which could be a barrier. Some degree of goal ambiguity might be useful at the supranational level, as it allows for politically acceptable framing and the continuation of the scheme's funding, but it can also pose a potential barrier to design and implementation at the national and school levels. |
| **Barriers and facilitators to the adoption of physical activity policies in elementary schools in Baden-Württemberg, Germany** (2) | In 2021, a cross-sectional study investigated the adoption of physical activity policies in elementary schools in Baden-Württemberg, Germany. An online questionnaire informed by the CFIR framework collected data on school and individual characteristics, which were then analysed using logistic regression to assess determinants of policy adoption. **Primary data collection was done following a cross-sectional design. Elementary and special needs school principals (n = 2,838) were invited to participate in the study.** In total, 121 schools (4%) participated in the survey, of which 49 (40.5%) reported having adopted a policy to promote physical activity. Positive associations with policy adoption were found for general willingness among teaching staff, access to available resources, access to knowledge and information, and stakeholder engagement. |
| **Compliance with tobacco control measures in Khyber Pakhtunkhwa, Pakistan** (3-5) | A mixed-methods design was used to assess Naswar sales and tobacco control compliance in Khyber Pakhtunkhwa, Pakistan. This included a cross-sectional observational study targeting Naswar vendors, utilising a multi-stage cluster sampling approach across three key districts: Peshawar, Mardan, and Bannu. The qualitative component, based on thematic analysis in MAXQDA, further explored barriers and facilitators through interviews with vendors and focus groups with tobacco farmers. The study's procedures complied with KMU's ethical oversight in Pakistan. Primary data collection was done following this design over two years. It is structured as a cross-sectional observational audit of shops selling smokeless tobacco (SLT), specifically Naswar, in three districts of the Khyber Pakhtunkhwa province (Peshawar, Mardan, and Bannu). The districts were chosen due to their significant Naswar consumption, production, and business scale. Two types of shops were observed: grocery and convenience stores (GPOS vendors) and wholesale dealers and manufacturers (ENS vendors). The eligibility criterion for all shops was that they sell at least one brand of Naswar. Shops in rural and urban areas were included. The quantitative component involved recruiting Naswar vendors based on shop density, with a calculated sample size of 273 shops: 50 in Bannu, 90 in Mardan, and 160 in Peshawar. Sampling was divided between GPOS, systematically selecting every third shop, and ENS shops, which were recruited due to their smaller numbers. The quantitative survey featured face-to-face interviews using a pretested, paper-based questionnaire covering Naswar sales, business duration, profit margins, awareness of health risks, and willingness to switch from Naswar sales. Logistic regression analyses were conducted to assess associations between various business factors and the willingness to stop selling Naswar. For the qualitative component, the study purposively selected participants through snowball sampling, using the concept of "information power" to achieve sample specificity and depth. Two groups were involved: SLT POS vendors and exclusive Naswar sellers, suppliers, wholesale dealers, and manufacturers. Additionally, Tobacco farmers from Swabi and Mardan were interviewed using focus group discussions (FGD) and in-depth interviews, conducted in Pashtu, recorded, transcribed, and analysed thematically in MAXQDA. Relevant FCTC articles informed the topic guide, which was piloted before use. Themes included barriers, facilitators, and perceptions of alternative livelihoods. Findings from both quantitative and qualitative approaches were synthesised to understand why Naswar supply chain actors continued in the business, their willingness to shift, and influencing factors. The qualitative data provided deeper insights into the challenges and motivations of vendors and farmers, while the survey offered broader trends and statistical associations. |

References

1.	Meshkovska B, Forberger S, Wendt J, Scheller DA, Castellari E, Tiboldo G, et al. Barriers and Facilitators to Implementation of the EU School Fruit and Vegetables Scheme: Cross Country Study. Global Implementation Research and Applications. 2023.

2.	Wendt J, Scheller DA, Flechtner-Mors M, Meshkovska B, Luszczynska A, Lien N, et al. Barriers and facilitators to the adoption of physical activity policies in elementary schools from the perspective of principals: An application of the consolidated framework for implementation research–A cross-sectional study. Frontiers in Public Health. 2023;Volume 11 - 2023.

3.	Ahmad F, Boeckmann M, Khan Z, Zeeb H, Khan MN, Ullah S, et al. Implementing smokeless tobacco control policy in Pakistan: a qualitative study among Naswar supply chain actors. Tobacco Control. 2021;30(5):548-52.

4.	Ahmad F, Khan Z, Boeckmann M, Khan MN, Siddiqi K, Ullah S, et al. Supply Chain Actors’ Willingness to Switch the Business of Smokeless Tobacco: A Mixed-Methods Study on Naswar Business in Pakistan. Nicotine & Tobacco Research. 2021;23(9):1498-506.

5.	Ahmad F, Khan Z, Siddiqi K, Khan MN, Boeckman M, Dregger S, et al. Compliance of Oral Snuff (Naswar) Packaging and Sales Practices with National Tobacco Control Laws and the Relevant Articles of Framework Convention on Tobacco Control in Khyber Pakhtunkhwa Pakistan. Nicotine & Tobacco Research. 2020;22(12):2224-30.
