# Supplementary material for: Bridging policy and implementation science: operationalising political and structural determinants for health policy implementation evaluation
Source: Implement Sci Commun. 2026 Oct 11;7:202. doi: 10.1186/s43058-026-01137-0 (PMC13678997; doi:10.1186/s43058-026-01137-0)
Supplement: Supplementary file 2 [file 43058_2026_1137_MOESM2_ESM.docx]

## Appendix 2: Predefined data extraction form with categories of the extracted data and characteristics of the categories

Table 1: Predefined data extraction form with categories of the extracted data and characteristics of the categories (1)

| **Type of Data** | **No.** | **Category** | **Characteristics** |  |
| --- | --- | --- | --- | --- |
| Metadata of publication | 1 | Author | Authors of the publication |  |
|  | 2 | Year | Year of publication |  |
|  | 3 | Title | Title of the paper |  |
|  | 4 | Journal | Name of the journal/publication medium |  |
|  | 5 | Publication type | Journal article, report, grey literature, theses, book |  |
| Metadata of public policy | 6 | Domain/Subdomain (Jurisdiction) | EU, national, regional, City level |  |
|  | 7 | Origin | Country of origin |  |
|  | 8 | Health policy field | SSB, PA, active transport with further specification if mentioned |  |
|  | 9 | Author of the policy | Name of the public body |  |
|  | 10 | Date of effect of the policy mentioned | If indicated |  |
|  | 11 | Date of revision or information about the history/evolution of the policy | - If a revision took place - If the policy is built upon previous policies/programmes/interventions (if the policy has a history) (2, 3) |  |
|  | 12 | Date of end of validity | If indicated |  |
|  | 13 | Aim of the policy | Specified in the publication |  |
| Data of the study | 14 | Study design | Design used for analysing the policy |  |
|  | 15 | Theoretical approach | Theory, framework, and model used within the publication |  |
| Policy/intervention | 16 | Operationalisation | Is the policy operationalised as one |  |
|  | 17 | Dimension of the implementation process | (a) Implementation structure (4-6)     - Type and organisations involved in the process     - Organizational setting (horizontal vs vertical, multilevel (how many levels and which), cross-sectional, private actor involvement)     - Policy-relates rules, procedures, resource allocation (b) Agency decision–making (transfer/operationalisation of legal stipulation in operational programmes/interventions) (6, 7)     - who within the process decided on delivery mode, leeway for discretion, scope and nature of involved personnel     - information about who guides   and drives the process   - position of the actors involved (supportive, rejecting, neutral, mixed) (8-10) - information about the organisation of the process  (c) Role of the target group within the implementation process, e.g. filling out requests, complying with conditions attached to benefits, comply with special treatment programmes (11, 12)   Policy results per intervention and for the whole policy (13) | |
|  | 18 | Further variables mentioned/analysed and their operationalisation | Further variables mentioned | |
|  | 19 | Evaluation | Is an evaluation planned for the policy? Was there an evaluation of the policy? | |
|  | 20 | Outcome/Impact | Result of the policy | |

References:

1.	Forberger S, Reisch L, Lien N, Meshkovska B, Lobczowska K, Scheller DA, et al. Analyzing public health policy implementation processes – a systematic map. <https://osf.io/7w84q/>: Open Science Foundation; 2020 [

2.	Greer SL. Choosing paths in European Union health services policy: a political analysis of a critical juncture. Journal of European Social Policy. 2008;18(3):219-31.

3.	Weible CM, Carter DP. Advancing Policy Process Research at Its Overlap with Public Management Scholarship and Nonprofit and Voluntary Action Studies. Policy Studies Journal. 2017;45(1):22-49.

4.	Bolleyer N, Börzel TA. Non-hierarchical policy coordination in multilevel systems. European Political Science Review. 2010;2(2):157-85.

5.	Thomann E, Lieberherr E, Ingold K. Torn between state and market: Private policy implementation and conflicting institutional logics*. Policy and Society. 2016;35(1):57-69.

6.	Tosum J, Treib O. Linking Policy Design and Implementation Styles In: Howlett M, Mukherjee I, editors. Routledge Handbook of Policy Design. New York: Routledge; 2018. p. 316-30.

7.	Sager F, Thomann E, Zollinger C, van der Heiden N, Mavrot C. Street-level Bureaucrats and New Modes of Governance: How conflicting roles affect the implementation of the Swiss Ordinance on Veterinary Medicinal Products. Public Management Review. 2014;16(4):481-502.

8.	Haverland M. National Adaptation to European Integration: The Importance of Institutional Veto Points. Journal of Public Policy. 2000;20(1):83-103.

9.	Steunenberg B. A policy solution to the European Union's transposition puzzle: Interaction of interests in different domestic arenas. West European Politics. 2007;30(1):23-49.

10.	Tsebelis G. Princeton: Princeton University Press; 2003.

11.	Howlett M. What is a Policy Instrument? Policy Tools, Policy Mixes, and Policy-Implementation Styles. In: Eliadis P, Hill MM, Howlett M, editors. Designing Government From Instruments to Governance. Montreal&Kingston, London, Ithaca: McGill-Queen's University Press; 2005. p. 21-30.

12.	Pierce JJ, Siddiki S, Jones MD, Schumacher K, Pattison A, Peterson H. Social Construction and Policy Design: A Review of Past Applications. Policy Studies Journal. 2014;42(1):1-29.

13.	Mettler S, SoRelle M. Policy feedback theory. In: Weibel CM, Sabatier PA, editors. Theories of the Policy Process. 4ed. ed. New York: Routledge; 2018. p. 151-81.
